# A Narrative Review on How Timing Matters: Circadian and Sleep Influences on Influenza Vaccine Induced Immunity

**DOI:** 10.3390/vaccines13080845

**Published:** 2025-08-08

**Authors:** Achilleas Livieratos, Jamie M. Zeitzer, Sotirios Tsiodras

**Affiliations:** 1 Independent Researcher, 15238 Athens, Greece; 2 Department of Psychiatry & Behavioral Sciences, School of Medicine, Stanford University, Stanford, CA 94305, USA; jzeitzer@stanford.edu; 3 4th Department of Internal Medicine, Attikon University Hospital, 12462 Athens, Greece; tsiodras@med.uoa.gr

**Keywords:** circadian, sleep, vaccination

## Abstract

We aimed to synthesize and critically evaluate human studies on the impact of circadian and sleep factors on influenza vaccine-induced immune responses. A comprehensive literature review was conducted, and of the 1260 studies identified, 13 met the inclusion criteria for evaluating vaccination timing, circadian misalignment, and sleep parameters in relation to influenza vaccine-induced immune responses in human populations. Most studies assessed humoral immune responses, primarily antibody titers. Morning vaccination (typically between 9:00 and 11:00 AM) was associated with higher antibody titers compared to afternoon vaccination, particularly for the A/H1N1 strain in adults aged ≥ 65 years. Short sleep duration—especially in the two nights preceding vaccination—was associated with reduced antibody levels, while acute sleep deprivation the night after vaccination transiently reduced antibody levels in males. Sleep fragmentation and excessive daytime sleepiness were linked to increased vulnerability to breakthrough infections. Evidence on circadian misalignment from shift work was mixed. Clinical outcomes were reported in one large trial, where morning vaccination correlated with fewer respiratory hospitalizations. Current evidence supports a potential role for circadian timing and sleep duration in enhancing vaccine-induced antibody responses, particularly in older adults and individuals with sleep or circadian disruption. However, inconsistencies, modest effect sizes, and methodological limitations preclude broad recommendations. Future studies should incorporate direct measures of circadian phase, stratify by chronotype and population (e.g., shift workers), and evaluate both immunologic and clinical outcomes to inform targeted chrono-immunization strategies.

## 1. Introduction

Circadian rhythms—endogenous 24-h cycles regulating physiology and behavior—are increasingly recognized as important modulators of immune function and vaccine responses [1,2,3,4]. Core immune processes such as leukocyte trafficking and cytokine secretion exhibit circadian variation [5,6]. Aligning vaccine administration with circadian biology has been proposed to optimize immunogenicity, especially in vulnerable populations [1,2,3,4].

Circadian disruption—arising from shift work, irregular sleep timing, or social jet lag—can blunt the amplitude of these rhythms and impair vaccine responses [7,8,9]. Sleep disturbances, which often accompany circadian misalignment, have also been linked to reduced antibody production post-vaccination [1,2,3,4]. Although the effects of circadian misalignment and poor sleep are well described mechanistically, human studies evaluating their impact on vaccine-induced immunity remain limited and heterogeneous.

Lifestyle factors (e.g., work schedules, caregiving duties) and methodological variability (e.g., sampling times, vaccine types, outcome definitions) also influence observed vaccine responses. Nevertheless, a growing body of evidence suggests that morning vaccination may enhance antibody production [10,11]. Emerging evidence indicates that this diurnal effect is most pronounced in older adults, where propensity for circadian misalignment may render early-morning vaccination a particularly effective strategy for maximizing their antibody responses in a setting of immunosenescence [11].

Sleep duration and quality, circadian rhythms, and exposure to light form an interconnected regulatory network that shapes immune function [2,3]. Sleep delivers essential restoration for antigen processing and T-cell priming, while endogenous circadian clocks orchestrate daily oscillations in leukocyte trafficking and cytokine secretion [5,6]. Light acts as the primary environmental zeitgeber, aligning these internal rhythms to the external day–night cycle via the suprachiasmatic nucleus [2,9]. Disruption at any node—insufficient or fragmented sleep, circadian misalignment, or inadequate daytime light exposure—can impair vaccine responses by altering immune-enhancing processes at both the cellular and molecular levels [10,11].

Given the interplay between circadian, sleep health, and immunologic response, chrono-optimization of vaccination has emerged as a promising area of investigation. Such strategies may hold particular relevance for older adults, shift workers, or those with sleep disorders. In this narrative review, we synthesize evidence from human studies examining circadian and sleep influences specifically on influenza vaccine-induced immunity. Due to the heterogeneity of study designs, populations, and measured outcomes, a narrative rather than a systematic review approach was selected to critically appraise existing evidence and identify gaps for future research.

Key Definitions [2,3,4,5]: Chronotype refers to an individual’s inherent preference for the timing of sleep and wake, influenced by genetic and environmental factors. Zeitgeber refers to any external cue—most notably light—that synchronizes endogenous circadian rhythms to external stimuli. Circadian misalignment refers to a state in which internal 24-h biological clocks are out of sync with the external environment, as occurs in jet lag or night-shift work.

## 2. Materials and Methods

We conducted a comprehensive literature search to identify studies published from January 1995 until June 2025 evaluating the impact of circadian timing and sleep on influenza vaccine-induced immune responses in human populations. Search strings combined influenza vaccination terms with circadian, sleep, light, and shift work descriptors and immunological outcome terms. The final PubMed syntax was (influenza AND vaccin*) AND (circadian OR time of day OR chronotype OR sleep OR insomnia OR sleep apnoea OR hypersomnia OR shift work OR light) AND (immune* OR antibody OR titer OR immunogenicity). Identical descriptors were adapted for Scopus and Embase.

This broader search, utilizing wildcard terms (*), returned more inclusive results, allowing us to capture a wider array of relevant studies on vaccine-induced immune responses in relation to sleep and circadian factors. Articles were screened for relevance based on the following inclusion criteria: human participants; assessment of circadian or sleep patterns or shift work in relation to vaccination, and reporting of immune outcomes (e.g., antibody titers, cytokine levels, or clinical endpoints). Exclusion criteria included animal or in vitro studies; non-English language publications; and studies unrelated to vaccine timing or immunologic response.

**Figure 1:**
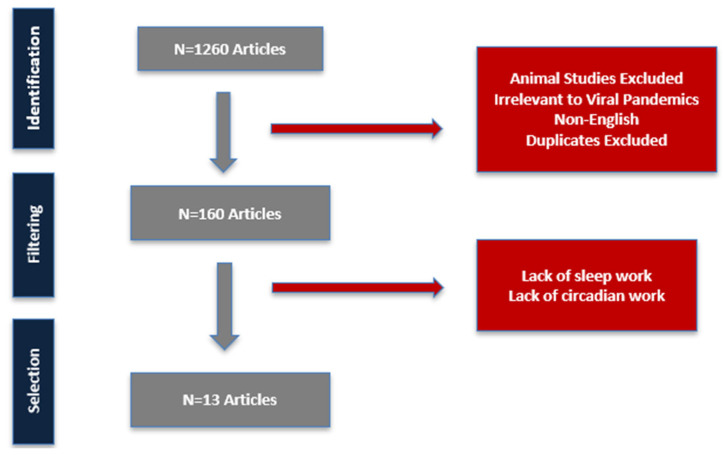
PRISMA-style flow diagram representing the study selection process.

Although this is a narrative review, we adapted elements of the PRISMA approach to improve transparency. More specifically, studies were excluded if they (i) did not evaluate influenza vaccination; (ii) lacked a sleep or circadian exposure of interest (defined as short sleep ≤ 6 h, sleep deprivation, shift work, irregular sleep timing, or documented circadian misalignment); or (iii) reported only animal or in vitro data. Irrelevant topics included basic immunology without vaccination, vaccine hesitancy, or unrelated clinical studies. A structured search, explicit inclusion/exclusion criteria, and a PRISMA-style flow diagram (Figure 1) were used to guide study selection [12,13,14,15,16,17,18,19,20,21,22,23,24].

After removing duplicates and excluding 1100 irrelevant articles, 160 full-text articles were reviewed. Of these, 149 were excluded for not directly analyzing the effect of circadian, or sleep factors on vaccine-induced immune response. A total of 13 studies met all inclusion criteria and were selected for qualitative analysis.

Each included study was independently reviewed by two investigators. Data were extracted on study design and methodology, participant characteristics, vaccine type and administration timing, measured immune outcomes (e.g., antibody titers, T-cell activity) and sleep or circadian-related exposures (e.g., sleep duration, circadian misalignment). Discrepancies between reviewers were resolved through discussion to ensure consensus.

Given the heterogeneity of study designs, exposure definitions, populations, and outcome measures, a narrative synthesis approach rather than a meta-analysis was employed. Patterns, trends, and contradictions were qualitatively assessed to highlight areas of inconsistency and knowledge gaps relevant to chrono-immunology and vaccine optimization.

Several additional exploratory studies, including investigations on SARS-CoV-2 vaccines, hepatitis A, and meningococcal conjugate vaccines, were reviewed to enrich only mechanistic context for circadian and sleep effects on vaccine responses. These were primarily identified through the same database searches, such as PubMed [25,26,27,28,29,30,31,32,33,34,35,36,37,38,39,40,41,42,43,44,45,46].

## 3. Results

We hereby summarize the key design features and participant characteristics of the 13 clinical and observational studies filtered, evaluating how time of day, sleep patterns, and related interventions influence humoral and cellular responses to influenza vaccines (Table 1). For each study, we report country and study setting, age distribution and gender balance, overall design and sample size, vaccination and follow-up periods, vaccine formulations, and any special population or procedural details (e.g., light exposure, melatonin supplementation, sleep deprivation).

### 3.1. Effects of Time-of-Day Vaccination on Immunogenicity

Across the eight studies that directly compared morning with afternoon or evening influenza vaccination, a consistent—though not universal—pattern was observed: antibody rises were typically stronger when the shot was given in the first half of the day; the signal was clearest in adults ≥65 years (Table 2). Four randomized or cluster-randomized trials and two large observational cohorts showed roughly 10–30% larger geometric mean titer increases after morning vaccination; the advantage was most reliable for the A/H1N1 strain (Table 2). Sex-stratified analyses indicated that the effect may be more pronounced in men [16]. Importantly, two well-designed studies detected no diurnal difference, underscoring that baseline immunity, strain characteristics, and self-selected clinic slots can mask a true timing effect [18,19]. Beyond surrogate markers, one post hoc analysis of more than 12,000 vaccinees linked earlier vaccination to fewer respiratory hospitalizations during the influenza season [12]. Taken together, current evidence supports the clinical value of scheduling routine influenza vaccination for the morning, particularly in the elderly.

### 3.2. Sleep Findings

Sleep duration in the nights immediately preceding vaccination has emerged as a key determinant of vaccine-induced antibody production (Table 3). Two studies used objective sleep assessment (polysomnography or wrist actigraphy), including one with experimentally enforced sleep deprivation, while the remaining relied on self-reported diaries or questionnaires [20,21,22,23,24]. In one study of 83 healthy young adults, shorter sleep during the two nights prior to immunization was significantly correlated with reduced antibody levels against the A/H1N1 (New Caledonia) strain at one and four months [21]. In contrast, sleep efficiency and subjective sleep quality did not significantly correlate with antibody levels [21].

Similarly, in a randomized trial, Benedict C et al. reported that total sleep deprivation the night after monovalent A/H1N1 immunization reduced antibody levels in males by ~60% on day five post-vaccination [22]. However, this effect was transient, with antibody levels normalizing by day 17. Studies on sleep deprivation and vaccine responses elicited by an A/H1N1 influenza vaccine demonstrated the critical role of sleep in early adaptive immune activation [7,29]. Men who were sleep-deprived the night following vaccination exhibited approximately 60% lower antibody titers five days post-vaccination compared to those who had normal sleep [7,29]. However, this impairment was transient; antibody levels in sleep-deprived males eventually caught up with those in the well-rested group by days 10, 17, and 52 post-vaccination [7,29]. Interestingly, no significant effect of sleep deprivation was observed in females, suggesting potential sex-based differences in immune responses [7,29].

Limited data were available for excessive daytime sleepiness. In one study, older men with excessive daytime sleepiness had significantly lower antibody titers to the influenza A/H3N2 strain 28 days post-vaccination with a trivalent vaccine compared to those with regular sleep patterns, suggesting even minor sleep disruptions can impair vaccine-induced immune response [23].

Chronic insomnia has also been associated with diminished humoral responses [24]. Taylor D.J. et al. reported that patients with chronic insomnia had significantly weaker humoral response four weeks post-influenza vaccination, potentially due to persistently elevated inflammatory markers [24].

### 3.3. Light Exposure and Circadian Stability

Münch et al. found that higher light exposure in institutionalized older adults was associated with increased antibody titers to influenza vaccination (Table 4), suggesting a role for environmental light in supporting circadian stability and vaccine response [15]. Patients with higher daily light exposure demonstrated significantly greater antibody responses to the H3N2 strain compared to those with lower exposure [15].

### 3.4. Circadian Misalignment and Shift Work

A small number of exploratory studies in non-influenza platforms—including meningococcal conjugate, hepatitis A, and SARS-CoV-2 vaccines—have reported circadian and sleep effects (e.g., stronger morning antibody responses, sleep-related titer modulation) analogous to those seen with influenza [25,26,31,40,45]. These findings suggest potential cross-vaccine chronobiological phenomena but fall outside our targeted influenza analysis and are very briefly summarized solely for context.

We identified mixed results regarding the effects of circadian misalignment and shift work on vaccine responses. One study found no significant differences in antibody titers between shift workers and daytime workers but did observe higher T-cell responses in night-shift workers [40]. In a proof-of-concept study of another vaccine targeting meningococcus type C (MenC), night-shift workers who slept ~1.8 h less per 24 h and exhibited a pronounced circadian phase delay mounted 25–35% smaller increases in MenC-specific IgG subclasses compared with day workers [45].

### 3.5. Clinical Outcomes

Very few studies reported data on clinical outcomes (Table 5), and these were mostly performed in the COVID-19 pandemic era. Evidence from COVID-19 vaccinations highlights that circadian misalignment or sleep fragmentation may increase hospitalization rates such that those individuals with weaker circadian rhythms exhibit reduced humoral response and also show heightened inflammatory markers, that could worsen disease severity if infected [25,26,31].

## 4. Discussion

The reviewed studies provide preliminary evidence suggesting an inter-relationship between circadian and sleep parameters on influenza vaccine-induced immunity (Table 6). However, the effects are neither consistent across all studies nor uniform across influenza vaccine strains. We attempted to separate the reviewed evidence and discuss its implications for future evaluations of these associations.

### 4.1. Mechanistic Basis of Morning Vaccine-Induced Immune Response

Circadian regulation of immune function likely explains the enhanced response to morning vaccination. Studies indicate that antigen presentation, T-cell activation, and cytokine release (e.g., IFN-gamma) peak during morning (between 9:00 and 11:00 am), aligning with better immunogenicity [12,13,14,15,16,17]. This temporal alignment appears particularly advantageous for influenza vaccines, where rapid activation of both humoral and cellular immunity is crucial for establishing protection against seasonal strains.

Juxtaposing Wyse et al.’s heterogeneous study against the more uniform10–30% morning boost found in our included influenza studies suggests that time-of-day effects are driven less by chance and more by vaccine-specific biology—for instance, unique antigen-presentation routes or adjuvant formulations [25]. Ince et al. demonstrated this principle beyond respiratory vaccines [46].

The molecular underpinnings of these effects involve core clock genes (e.g., BMAL1, CLOCK) that regulate leukocyte trafficking and lymphoid tissue function [5,6]. While similar chronobiological principles may apply to other vaccine platforms, the unique virological characteristics of influenza—including rapid mutation rates and strain-specific antigenic drift—suggest these circadian interactions may be particularly consequential for annual influenza vaccination strategies.

### 4.2. Sleep Duration, Quality and Continuity

Adequate sleep before and shortly after vaccination supports early immune processes such as antigen processing and T-cell priming [7,29]. The transient nature of sleep deprivation effects underscores the importance of timing and duration of sleep rather than chronic effects [7,29]. While total sleep duration consistently affects immunogenicity, the role of sleep quality is less consistent. Fragmented sleep and excessive daytime sleepiness may impair immune responses, but not all studies agree, indicating a need for standardized sleep assessment in future research [23,24]. Fragmented sleep disrupts deep sleep stages essential for immune function, reducing the release of immune-enhancing cytokines [21,22].

Five studies employing actigraphy, polysomnography, or validated questionnaires reveal that shorter sleep in the nights preceding and following vaccination correlates with markedly lower antibody responses [20,21,22,23,24]. Chronic insomnia similarly was associated with significantly lower H3N2 and B hemagglutination inhibition titers at four weeks post-influenza vaccination compared to controls [23]. These data underscore peri-vaccination sleep as a modifiable determinant of influenza immunogenicity. Some of the discrepancies noted in the sleep studies indicate the need to carefully distinguish different aspects of sleep in evaluating their impact on vaccine-induced immune response. Nevertheless, sufficient and uninterrupted sleep is essential for optimal antigen processing, T-cell activation, and antibody production, further reinforcing the need to prioritize sleep health around vaccination. Importantly, a 2023 meta-analysis found that associations between short sleep and reduced antibody titers were significant only when sleep was measured objectively [43].

### 4.3. Light Exposure

Light exposure as a circadian stabilizer may enhance the vaccine response, particularly in individuals with neurological conditions like dementia. The association between higher light exposure and increased antibody titers in older adults supports this hypothesis and highlights the potential of non-pharmacologic circadian interventions [15].

### 4.4. Impact of Circadian Misalignment and Shift Work

The circadian system, governed by clock genes, modulates immune function via rhythmic regulation of cytokines and immune cell trafficking [5,6,27]. During the biological night, melatonin binds to MT1 and MT2 receptors on immune cells, enhancing anti-inflammatory responses and promoting tissue repair. Disruption of these rhythms—through insomnia, irregular schedules, or shift work—can impair vaccine responses by blunting the release of key cytokines like TNF-α and IFN-γ [5,6,27].

Circadian misalignment due to shift work does not consistently impair humoral vaccine responses, but emerging evidence suggests it may influence cellular immunity [37,38,39,40,45]. For instance, a few studies reported no differences in the humoral response between day- and night-shift workers, despite shorter sleep duration in the latter group [39,40].

These findings suggest that acute circadian misalignment might not uniformly suppress immune response and may differentially affect humoral versus cellular arms.

Taken together, the evidence highlights the complexity of circadian influences on vaccination responses and the need for controlled studies stratified by chronotype, exposure timing, and vaccine type.

### 4.5. Circadian Disruption and Vulnerable Populations

The impact of circadian misalignment on influenza vaccine responses presents substantial public health considerations that warrant careful examination across different at-risk groups. For older adults, the confluence of age-related circadian dysfunction and immunosenescence creates a particularly vulnerable physiological state. The aging process typically leads to dampened circadian amplitude, characterized by reduced melatonin secretion, flattened cortisol rhythms, and advanced sleep phase timing [11,25]. These changes coincide with the well-documented decline in immune function known as immunosenescence, which includes reduced vaccine responsiveness [11,25,27]. We report that the most robust and consistent morning vaccination benefits in studies specifically examining adults aged ≥65 years, with some trials reporting higher antibody titers when vaccines were administered before noon compared to afternoon administration [12,13,14,15,16,17,18,19]. This population may therefore derive particular benefit from chrono-optimized vaccination strategies.

The clinical implications of these findings are underscored by the single large-scale study that examined hard endpoints, which found morning influenza vaccination associated with a significant reduction in respiratory-related hospitalizations during the influenza season [12]. This suggests that circadian optimization strategies may potentially yield benefits extending beyond surrogate immunological markers to tangible improvements in clinical outcomes. Together, these observations highlight the need for targeted vaccination approaches in populations experiencing circadian disruption, whether due to aging, occupational demands, or other lifestyle factors.

### 4.6. Broad Vaccination Insights

Emerging evidence from other vaccination studies suggests circadian influences similar to influenza vaccines, with systematic reviews, trials, and observational cohorts demonstrating stronger antibody responses following morning vaccination and adequate peri-vaccination sleep, though effect sizes vary by population and vaccine platform [26,32,33,34,35,36]. However, direct comparisons with influenza vaccines remain limited by differences in immune pathways, viral evolution, and clinical outcomes, necessitating cautious interpretation of these parallel findings [47].

Some influenza vaccine trials report larger circadian or sleep-related effects for certain viral strains (e.g., H3N2 vs. H1N1) [12,13,14,15,16,17,18,19,20,21,22,23,24]. One possibility is that antigen presentation kinetics differ by strain or platform, so that clock-gated pathways (e.g., dendritic cell migration, melatonin modulation of Toll-like receptors) disproportionately influence the early priming of some antigens [4,9]. Alternatively, neutrophil and monocyte trafficking—which follows robust circadian rhythms—may interact differently with strain-specific adjuvants to amplify antibody output for certain formulations [6].

Older adults generally exhibit both blunted circadian amplitudes and poorer sleep quality [25,26,27]. In contrast, younger adults—who have higher circadian amplitude and sleep efficiency—may derive larger relative gains in vaccine response when their sleep/circadian rhythms are supported [5,7].

Women often mount stronger antibody responses than men, yet they also report more sleep disturbance and greater vulnerability to circadian misalignment during shift work or jet lag [3,7]. Sex hormones modulate both clock gene expression and sleep architecture, which could interact to produce sex-specific vaccine outcomes [7,10].

### 4.7. Limitations

Methodological limitations were common across the included studies. This included failure to adjust for multiple comparisons between influenza antigens, lack of control for baseline antibody levels, omission of randomization factors (e.g., primary care practice), and relatively small effect sizes with wide inter-individual variability, as reflected in broad confidence intervals. These issues underscore the need for caution when interpreting the reported effects of circadian timing and sleep on immune responses.

Concerns raised by Kurupati et al., particularly regarding confounding from blood sample timing, further illustrate the complexities in evaluating vaccination timing effects [21]. Antibody titer outcomes, while convenient, are often poor surrogates for vaccine effectiveness and show high inter-subject variability [44]. These limitations, along with contradictory findings, highlight the need for robust, well-designed studies before implementing broad recommendations on vaccination timing (see Table 2). Additionally, potential publication bias—favoring studies with positive outcomes—may overstate the true impact of circadian and sleep-related influences on vaccine-induced immunity.

A key limitation across many studies is the absence of objective circadian markers (e.g., dim-light melatonin onset). Reliance on local clock time alone means that nominal morning and evening groups may differ only slightly in external time yet be separated by 5–8 h in internal biological time, potentially obscuring phase-dependent effects [27]. This issue is particularly evident in shift work cohorts, where vaccination responses were stratified by work schedule (day vs. night) without assessing circadian phase or schedule duration, increasing the risk of false-negative results. While existing studies hint at the importance of sleep, light, and timing for vaccine responses, this remains a wide-open area for investigation. Very few trials to date employ objective, multidimensional measures of sleep alongside circadian phase markers or individual mid-sleep time. Nor have studies consistently anchored vaccine administration to each participant’s internal clock rather than arbitrary clock hours. Future work should therefore embed sleep and circadian metrics into trial design by timing inoculations relative to each subject’s circadian phase, extending follow-up windows longitudinally—from pre-vaccination baseline through acute post-vaccination and extended follow-up—to capture transient versus sustained immune effects, and testing these approaches across diverse vaccines, age groups, and chronotypes.

Similarly, the single bright-light intervention study reported improved antibody titers with higher daytime illuminance but did not measure intermediary sleep changes or markers of circadian entrainment, rendering the proposed mechanism speculative. More broadly, most studies relied on self-reported sleep and collected only one or two post-vaccination immunogenicity time points, limiting both measurement precision and temporal resolution.

Finally, as most studies were conducted in healthy adults in high-income settings, generalizability to pediatric populations, low-resource contexts, and vaccines other than influenza remains limited.

## 5. Conclusions

Emerging evidence suggests that circadian rhythms modulate vaccine-induced immune responses, with morning vaccinations often associated with stronger antibody production than afternoon doses. This effect appears most prominent in older adults, who experience immunosenescence—a progressive decline in immune function that may heighten vulnerability to circadian misalignment.

Sleep is another critical factor. Optimizing sleep duration and quality before and after vaccination may enhance immune responses. Interventional studies using strategies like pre-vaccination sleep extension or melatonin therapy could clarify whether improving sleep enhances responses—particularly in individuals with sleep disorders, insomnia, or age-related circadian dysfunction.

Psychological stress adds further complexity. Future research should evaluate whether stress reduction interventions, such as mindfulness or behavioral therapy, can improve vaccine outcomes by enhancing circadian and sleep regulation.

Future research should employ objective measurements of circadian phase and sleep (e.g., actigraphy, melatonin onset), stratify participants by chronotype, and assess both immunological and clinical endpoints. Interventional studies in diverse populations—including shift workers, older adults, and individuals in low-resource settings—are needed to establish causality. Evaluating the logistical feasibility and cost-effectiveness of timing-based vaccination strategies will also be essential for real-world implementation.

Finally, public health messaging must convey these findings with precision and care. Any recommendations regarding vaccine timing should be grounded in robust evidence, clearly distinguish timing effects from vaccine safety and efficacy, and avoid inadvertently contributing to vaccine hesitancy.

## Figures and Tables

**Table 1:** Descriptive characteristics of included studies investigating the effects of circadian timing and sleep on influenza vaccine-induced immunity.

Study	Country (Region)	Age Range/Mean Age	Gender Stratification	Study Design	N (Sample Size)	Study Period	Vaccine Type	Other Defining Characteristics
Christensen J et al., 2024 [12]	Denmark	65–79 yrs (mean 71.7 ± 3.9 yrs)	47.1% female (5 877/12,477)	Post hoc analysis of pragmatic, open-label RCT (QIV-HD vs. QIV-SD)	12,477	Vaccinations 1 Oct 2021–20 Nov 2021; FU to 31 May 2022	High-dose vs. standard-dose quadrivalent influenza	Vaccination times 07:05 AM–08:08 PM (median 11:29 AM); early vs. late groups by median; registry-based comorbidity capture; outcomes via passive registry follow-up
Liu Y et al., 2022 [13]	China	50–75 yrs (mean 62.8 ± 7.2 yrs)	37.5% male, 62.5% female	Individual RCT; morning (9–11 AM) vs. afternoon (3–5 PM) vaccination with stratified block randomization	418	Enrolment 27 Oct 2020–22 Dec 2020; FU at 1 mo and 3 mo post-vax	Trivalent inactivated influenza (Sanofi split virion: H1N1 pdm09, H3N2, B/Victoria)	Baseline blood draw 8–10 AM; subgroup by age and sex; no serious AEs
Long JE et al., 2016 [14]	UK (West Midlands)	≥65 yrs (mean 71.3 ± 5.5 yrs)	52.2% female overall	Cluster RCT: 24 GP surgeries randomized annually to morning (9–11 AM) vs. afternoon (3–5 PM); baseline and 1 mo blood draws	298 consented; 276 analyzed	Enrolment 28 Oct 2011–12 Nov 2013; FU Dec 2013	Trivalent inactivated seasonal influenza (2011/12–2013/14; Pfizer Enzira^®^, Sanofi, BGP, GSK, Janssen formulations)	No acute infection/immunosuppressants; combined H1N1/H3N2/B analysis
Münch M et al., 2022 [15]	Switzerland (Wetzikon, Zurich)	55–95 yrs (mean 78.3 ± 8.9 yrs)	67.5% female, 32.5% male	Cross-sectional: 8 wk light monitoring; split into high- vs. low-illuminance groups; vaccination wk 44; 4 wk post-vax titers	80	Fall/winter 2012; vaccination wk 44; 4-wk FU	Trivalent inactivated influenza (Fluarix^®^: H3N2, H1N1, IB; 2012/13 WHO)	Institutionalized dementia; median illuminance 392.7 lux; HAI assay; no serious AEs
Phillips AC et al., 2008 [16]	UK (Birmingham)	Study 1: 22.9 ± 3.9 yrs; Study 2: 73.1 ± 5.5 yrs	Study 1: 34 M/41 F; Study 2: 38 M/51 F	(1) RCT: hepatitis A (10–12 PM vs. 4–6 PM); (2) observational: influenza (8–11 AM vs. 1–4 PM); ~1 mo blood draws	75/89	Baseline and ~28–31 days post-vax	Trivalent influenza (2003–04: A/New Caledonia; A/Panama; B/Shangdong)	Predominantly White; enzyme immunoassay and HAI; men showed higher AM responses
Kurupati RK et al., 2017 [17]	USA (Durham,)	Younger: 30–40 yrs; elderly: ≥65 yrs	Not stratified	5 yr cohort: AM vs. PM grouping; VNA, IgG/IgM/IgA, B-cell subsets, transcriptomics from matched samples	59/80	2011–2015 seasons; pre-vax, days 7, 14/28 draws	Trivalent inactivated influenza (Fluarix™ trivalent or quadrivalent)	Community adults; higher baseline PM titers in elderly patients drove fold-response patterns
Langlois PH et al., 1995 [18]	USA (Princeton,)	Adult volunteers (N/A)	N/A	Field trials: summer 1984 and revaccination 1985 (Princeton); autumn 1985 (Houston); AM vs. PM injections; antibody and local reaction assessment	N/A	Summer 1984; revaccination 1985; autumn 1985	Trivalent inactivated influenza (A/Philippines; A/Chile; B/USSR)	Diurnal pattern for A/Philippines only; more local reactions PM
Faustini SE et al., 2025 [19]	UK (Birmingham)	> 50 yrs (mean 57.0 ± 6.7 yrs)	65.0% female, 35.0% male	Observational: self-selected AM (8–10 AM) vs. PM (4–6 PM); co-admin of PPV23+quadrivalent influenza; multiplex Luminex, HAI, cytokines, hsCRP, cortisol	140	Jun 2018–Feb 2020; seasons 2018–19 and 2019–20; FU to 52 wks	PPV23 (Pneumovax^®^) & quadrivalent influenza (2018–19 Sanofi); 2019–20 Flucelvax-tetra)	Healthy > 50; excluded recent PPV23 or current-year flu; no diurnal effect; sustained immunity at 1 yr
Lee RU et al., 2024 [20]	USA (Bethesda/Silver Spring, MD)	18–64 yrs (mean 38 ± 12 yrs)	50.9% female	Open-label RCT: melatonin (5 mg) vs. control for 14 days post-vax; HAI and FluoroSpot at 14–21 days	108 (53 M; 55 C)	Oct 2022–Jan 2023; FU at 14–21 days post-vax	FluLaval^®^ Quadrivalent 2022/23 (A/Victoria; A/Darwin; B/Austria; B/Phuket)	Melatonin 1 h pre-bed; increases IFN-γ + GzB double-secretor GMFR; no HAI differences
Prather A.A. et al., 2021 [21])	USA (multiple universities)	18–25 yrs (mean 18.3 ± 0.9 yrs)	55.4% female (46/83)	Prospective: 13 d sleep diaries; vaccination on day 3; HAI at baseline, 1 mo, 4 mo	83	Sep–Nov 2000 and 2001; 13 d monitoring; FU at 1 mo and 4 mo	Fluzone^®^ (A/New Caledonia; A/Panama; B/Yamanashi or B/Victoria)	College freshmen; shorter sleep pre-vax predicted lower titers
Benedict C et al., 2012 [22]	Sweden (Uppsala and Solna)	~ 20.5 yrs	54.2% female (13/24)	Randomized: 24 h sleep deprivation vs. normal sleep post-H1N1 (Pandemrix™); HAI at days 5, 10, 17, 52	24 (13 sleep; 11 SD)	Vaccination 27 Nov 2009; FU days 5, 10, 17, 52 (2010)	Pandemrix™ (H1N1)	Healthy students; reduced day 5 antibody in SD males only; no lasting difference
Quach H.Q. et al., 2023 [23]	USA (Rochester, MN)	≥ 65 yrs (median 71.3 yrs)	61.4% female (129/210)	Prospective cohort: HD Flu vs. MF59Flu; STOP/ESS/PSQI; HAI at day 0 and 28; questionnaires ~1 yr post-vax	210	Aug–Dec 2018; blood day 0 and 28; ~1 yr questionnaires	HD or adjuvanted trivalent influenza (A/H1N1; A/H3N2; B)	Male excessive daytime sleepiness lowers H3N2 titers at D0 and D28; no OSA or PSQI effects
Taylor D.J. et al., 2020 [24]	USA (Denton)	18–29 yrs (mean 20.24 ± 2.60 yrs)	60% female (80/133)	Repeated-measures cohort: chronic insomnia (n = 65) vs. no insomnia (n = 68); structured clinical interview; blood at baseline (12–2 PM) and 4 wk post-vax	133 (65 Ins; 68 NoIns)	Recruitment 2011 and 2012 seasons; blood draws Sep–early Nov	Trivalent inactivated influenza (2011/12: A/California/7/2009; A/Perth/16/2009; B/Brisbane/60/2008; 2012/13: A/California/7/2009; A/Victoria/361/2011; B/Wisconsin/1/2010)	Healthy young adults; rigorous insomnia diagnosis (SCID-I/II, ISI, PSQI, ESS, MEQ); blood drawn 12–2 PM to control diurnal; insomnia group had lower baseline and post-vax H3N2 and B titers; no Group/Time interaction

ESS, Epworth Sleepiness Scale; F, female; FU, follow-up; GMFR, geometric mean fold rise; GzB, granzyme B; HAI, haemagglutination inhibition; HD, high dose; hsCRP, high-sensitivity C-reactive protein; IFN γ, interferon gamma; IgA, immunoglobulin A; IgG, immunoglobulin G; IgM, immunoglobulin M; Ins, insomnia group; ISI, Insomnia Severity Index; M, male; MEQ, Morningness–Eveningness Questionnaire; MF59Flu, MF59-adjuvanted influenza vaccine; mo, month(s); N/A, not applicable or not available; NoIns, no insomnia group; OSA, obstructive sleep apnoea; PPV23, 23-valent pneumococcal polysaccharide vaccine; PSQI, Pittsburgh Sleep Quality Index; QIV HD, high dose quadrivalent influenza vaccine; QIV SD, standard dose quadrivalent influenza vaccine; RCT, randomized controlled trial; SCID-I/II, Structured Clinical Interview for DSM-IV Axis I/II Disorders; SD, sleep deprivation; STOP, STOP-BANG questionnaire for obstructive sleep apnoea risk; VNA, viral neutralization assay; wk(s), week(s); WHO, World Health Organization; yrs, years.

**Table 2:** Effects of time-of-day vaccination on immunogenicity.

Included Study	Primary Outcome	Times of Vaccination and Type of Vaccine	Findings	Sleep and Circadian Parameters	Contradictory Findings, Null Results, and Limitations
Liu Y et al., 2022, China [13]	Antibody titer responses to influenza vaccination	9–11 AM vs. 3–5 PM; trivalent inactivated influenza	In 65–75 yr subgroup, morning increased A/H1N1 (49.5 vs. 32.9; *p* = 0.050) and increased A/H3N2 (93.5 vs. 73.1; *p* = 0.021)	None reported	Overall null except subgroup signals; limited generalizability beyond older adults and women
Long JE et al., 2016, UK [14]	Change in antibody titers one month post-vaccination	9–11 AM vs. 3–5 PM; trivalent seasonal influenza	Morning slots enhanced A/H1N1 response (263.6, 95% CI –1.62 to 525.59; *p* = 0.05)	None assessed	Strain-specific effect; no A/H3N2 change; cluster design and non-blinding may affect inference
Phillips AC et al., 2008, UK [16]	Antibody titer response to influenza vaccine	8–11 AM vs. 1–4 PM; 2003–04 trivalent influenza	Men vaccinated AM had higher A/Panama GMT (F(1,84) = 5.93; *p* = 0.02); no effect in women	Diurnal rhythm inference only	Effects limited to men; small n; potential for unmeasured confounding
Kurupati RK et al., 2017, USA [17]	Antibody titer responses to influenza vaccination	8 AM–12 PM vs. 12–5 PM; Fluarix™ tri-/quadrivalent	In elderly group, morning increased H1N1 titers vs. PM; but higher baseline PM titers confounded fold-rise calculations (*p* < 0.05)	Timing of blood draw	Confounded by sample-collection time; limits clear assignment of vaccine-time effect
Langlois P.H. et al., 1995, USA [18]	Hemagglutination-inhibition titers (three flu strains)	Morning vs. afternoon IM injections across normal work hours	Princeton 1984: AM > PM for A/Philippines only; no effect for other strains or in revaccination cohorts	Clock-time of injection only	Strain- and year-specific; non-random time assignment; possible site/season confounding
Faustini S.E. et al., 2025, UK [19]	Pneumococcal IgG/IgA/IgM and influenza HAI titers over 1 year	AM (08:00–10:00) vs. PM (16:00–18:00); PPV-23 + quadrivalent influenza	Both groups increased pneumococcal and influenza titers; no time-of-day differences in magnitude or durability for any isotype/strain	Questionnaire on sleep duration, activity, diet; cortisol/cytokine profiling	Null contrasts earlier elderly findings; self-selected slots; younger cohort; co-administration may dilute effect; only two time windows tested

IgG/IgA/IgM, immunoglobulin G/A/M.

**Table 3:** Sleep findings.

Included Study	Primary Outcome	Intervention/Exposure and Vaccine	Findings	Sleep and Circadian Parameters	Contradictory Findings, Null Results, and Limitations
Benedict C et al., 2012, Sweden [22]	HAI titer response to H1N1 vaccination	Sleep 8 h vs. total sleep deprivation; Pandemrix™	SD males only showed lower day-5 HAI (60% lower; *p* ≤ 0.05); no lasting differences by day 10, 17, 52	24 h sleep deprivation	Small sample size; sex-specific transient effect; no sustained impact
Lee R.U. et al., 2024, USA [20]	Humoral (HAI) and cellular (FluoroSpot) responses	5 mg melatonin nightly × 14 days post-flu shot	No HAI differences; melatonin increased IFN-γ + GzB double-secretor GMFR at 14–21 days	Melatonin taken about57 min before sleep	Small pilot; only cellular endpoints positive; requires replication
Prather A.A. et al., 2021, USA [21]	HAI titers at 1 mo and 4 mo post-vaccination	13 days self-reported sleep diaries; trivalent influenza	Shorter sleep nights 2–3 pre-vax predicted ↓ 1 mo and 4 mo HAI titers; no effect of sleep efficiency or quality	Sleep duration, efficiency, quality via diary	Subjective reporting; small sample; no sleep quality effects
Quach H.Q. et al., 2023, USA [23]	HAI titers to A/H3N2 at Day 0 and 28	HDFlu or MF59Flu; ESS, STOP-BANG, PSQI	Excessive daytime sleepiness in men lowered H3N2 titers at D0 (*p* = 0.03) and D28 (*p* = 0.019); no effect in women; no OSA/sleep quality associations	ESS, STOP-BANG, PSQI; daytime sleepiness	Male-specific; observational/recall bias; cannot infer causation
Taylor D.J. et al., 2020, USA [24]	HAI titers at baseline and 4 wk post-vax	Chronic insomnia vs. no insomnia; trivalent influenza	Insomnia group ↓ baseline and 4 wk H3N2 (*p* = 0.008) and B (*p* = 0.009) titers; no Group andTime interaction	SCID, ISI, PSQI, ESS, MEQ; sleep diaries	Healthy young cohort; subjective measures; no analysis of acute sleep deprivation

ESS, Epworth Sleepiness Scale; GMFR, geometric mean fold rise; GzB, granzyme B; HAI, haemagglutination inhibition; HD, high dose; hsCRP, high-sensitivity C-reactive protein; IFN γ, interferon gamma; Ins, insomnia group; ISI, Insomnia Severity Index; M, male; MEQ, Morningness–Eveningness Questionnaire; mo, month(s); N/A, not applicable or not available; NoIns, no insomnia group; OSA, obstructive sleep apnoea; PSQI, Pittsburgh Sleep Quality Index; SCID-I/II, Structured Clinical Interview for DSM-IV Axis I/II Disorders; SD, sleep deprivation; STOP, STOP-BANG questionnaire for obstructive sleep apnoea risk.

**Table 4:** Light exposure and circadian stability.

Included Study	Primary Outcome	Exposure and Vaccine	Findings	Sleep and Circadian Parameters	Contradictory Findings, Null Results, and Limitations
Münch M et al., 2022, Switzerland [15]	HAI titers to H1N1, H3N2, IB	High vs. low daily light; Fluarix^®^ seasonal influenza	High-light group increased H3N2 GMT by ratio 9.9 (95% CI 3.2; *p* = 0.01) vs. low-light; no serious AEs	Higher light increased relative amplitude and inter-daily stability of rest and activity	Preliminary in dementia; small n; needs replication

GMT, geometric mean titre; AE, adverse event.

**Table 5:** Clinical outcomes.

Included Study	Primary Outcome	Times of Vaccination and Type of Vaccine	Findings	Sleep and Circadian Parameters	Contradictory Findings, Null Results, and Limitations
Christensen J et al., 2024, Denmark [12]	Incidence of respiratory hospitalizations and allcause mortality	7:05 AM–8:08 PM; QIV-HD vs. QIV-SD; early vs. late (median 11:29 AM)	Early vaccination leads to fewer respiratory hospitalizations; QIV-HD decreased pneumonia/influenza admissions (IRR 0.30; 95% CI 0.14–0.64)	Explored circadian timing; no sleep outcomes directly measured	Exploratory post hoc; observational nature; no time/vaccine type interaction; needs confirmation

QIV HD, high dose quadrivalent influenza vaccine; QIV SD, standard dose quadrivalent influenza vaccine; IRR, incidence rate ratio.

**Table 6:** Impact of circadian and sleep disruption on vaccine outcomes.

Section	Parameter	Impact on Vaccine Outcome	Key Findings
Effects of Time-of-Day Vaccination	Time of Vaccination	Morning vaccinations yield stronger immune responses, especially in older adults	Morning shots generally produce higher antibody titers—particularly in older subgroups—but effect size and strain specificity vary (e.g., no significant difference for influenza A/H3N2 in some trials).
Sleep Findings	Sleep Duration	Shorter sleep duration reduces vaccine-induced antibody responses	Consistent evidence shows that shorter sleep—especially the 1–2 nights before vaccination—significantly impairs antibody production. Acute 24 h deprivation causes transient titer drops that recover by day 10–17.
	Sleep Quality	Poor sleep quality (fragmented sleep) may compromise vaccine-induced immunity	Findings are mixed: some studies link fragmented sleep to greater post-vaccine infection risk, but not all show significant antibody-titer reductions.
	Chronic Insomnia	Chronic insomnia and fragmented sleep may impair vaccine responses	Insomnia cohorts often have lower baseline and post-vax titers for select strains; sleep fragmentation more clearly impacts cellular markers. Melatonin boosts cellular responses but not consistently antibody titers.
Light Exposure and Circadian Stability	Light Exposure	Higher daily light exposure enhances antibody responses via improved circadian stability	Preliminary data in institutionalized dementia patients show high-light groups mount significantly greater H3N2 titers at 4 weeks—likely via strengthened rest-activity rhythms.
Circadian Misalignment and Shift Work	Circadian Disruption	Immune response may be blunted in individuals with disrupted rhythms (e.g., shift workers)	Evidence is mixed: some report reduced antibody responses under misalignment, while others find no humoral change but altered cellular markers in night-shift groups. Older adults appear particularly vulnerable to misalignment, compounding immunosenescence.
Clinical Outcomes	Clinical Endpoints	Morning vaccination linked to reduced hospitalizations and mortality	Early quadrivalent influenza vaccination was associated with fewer respiratory hospitalizations and lower pneumonia/influenza admissions but findings are exploratory.

## Data Availability

No new data were created or analyzed in this study. Data sharing is not applicable to this article.

## References

[1] Zimmermann P., Curtis N. (2019). Factors That Influence the Immune Response to Vaccination. Clin. Microbiol. Rev..

[2] Ding J., Chen P., Qi C. (2024). Circadian rhythm regulation in the immune system. Immunology.

[3] Haspel J.A., Anafi R., Brown M.K., Cermakian N., Depner C., Desplats P., Gelman A.E., Haack M., Jelic S., Kim B.S. (2020). Perfect timing: Circadian rhythms, sleep, and immunity—An NIH workshop summary. JCI Insight.

[4] Cermakian N., Stegeman S.K., Tekade K., Labrecque N. (2022). Circadian rhythms in adaptive immunity and vaccination. Semin. Immunopathol..

[5] Downton P., Early J.O., Gibbs J.E. (2020). Circadian rhythms in adaptive immunity. Immunology.

[6] Reglero-Real N., Rolas L., Nourshargh S. (2019). Leukocyte Trafficking: Time to Take Time Seriously. Immunity.

[7] Spiegel K., Rey A.E., Cheylus A., Ayling K., Benedict C., Lange T., Prather A.A., Taylor D.J., Irwin M.R., Van Cauter E. (2023). A meta-analysis of the associations between insufficient sleep duration and antibody response to vaccination. Curr. Biol..

[8] Tauman R., Henig O., Rosenberg E., Marudi O., Dunietz T.M., Grandner M.A., Spitzer A., Zeltser D., Mizrahi M., Sprecher E. (2024). Relationship among sleep, work features, and SARS-cov-2 vaccine antibody response in hospital workers. Sleep Med..

[9] Walch O., Tavella F., Zeitzer J.M., Lok R. (2025). Beyond phase shifting: Targeting circadian amplitude for light interventions in humans. Sleep.

[10] Kim E.S., Oh C.E. (2022). Sleep and vaccine administration time as factors influencing vaccine immunogenicity. KMJ.

[11] Vink K., Kusters J., Wallinga J. (2025). Chrono-optimizing vaccine administration: A systematic review and meta-analysis. Front. Public Health.

[12] Christensen J., Johansen N.D., Janstrup K.H., Modin D., Skaarup K.G., Nealon J., Samson S., Loiacono M., Harris R., Larsen C.S. (2024). Time of day for vaccination, outcomes, and relative effectiveness of high-dose vs. standard-dose quadrivalent influenza vaccine: A post hoc analysis of the DANFLU-1 randomized clinical trial. J. Infect..

[13] Liu Y., Zhang H., Yuan G., Yao M., Li B., Chen J., Fan Y., Mo R., Lai F., Chen X. (2022). The impact of circadian rhythms on the immune response to influenza vaccination in middle-aged and older adults (IMPROVE): A randomised controlled trial. Immun. Ageing.

[14] Long J.E., Drayson M.T., Taylor A.E., Toellner K.M., Lord J.M., Phillips A.C. (2016). Corrigendum to ‘Morning vaccination enhances antibody response over afternoon vaccination: A cluster-randomised trial’ [Vaccine 34 (2016) 2679–2685]. Vaccine.

[15] Münch M., Goldbach R., Zumstein N., Vonmoos P., Scartezzini J.L., Wirz-Justice A., Cajochen C. (2022). Preliminary evidence that daily light exposure enhances the antibody response to influenza vaccination in patients with dementia. Brain Behav. Immun. Health.

[16] Phillips A.C., Gallagher S., Carroll D., Drayson M. (2008). Preliminary evidence that morning vaccination is associated with an enhanced antibody response in men. Psychophysiology.

[17] Kurupati R.K., Kossenkoff A., Kannan S., Haut L.H., Doyle S., Yin X., Schmader K.E., Liu Q., Showe L., Ertl H.C.J. (2017). The effect of timing of influenza vaccination and sample collection on antibody titers and responses in the aged. Vaccine.

[18] Langlois P.H., Smolensky M.H., Glezen W.P., Keitel W.A. (1995). Diurnal Variation in Responses to Influenza Vaccine. Chronobiol. Int..

[19] Faustini S.E., Backhouse C., Duggal N.A., Toellner K.M., Harvey R., Drayson M.T., Lord J.M., Richter A.G. (2025). Time of day of vaccination does not influence antibody responses to pneumococcal and annual influenza vaccination in a cohort of healthy older adults. Vaccine.

[20] Lee R.U., Watson N.L., Glickman G.L., White L., Isidean S.D., Porter C.K., Hollis-Perry M., Walther S.R., Maiolatesi S., Sedegah M. (2024). A randomized clinical trial of the impact of melatonin on influenza vaccine: Outcomes from the melatonin and vaccine response immunity and chronobiology study (MAVRICS). Hum. Vaccines Immunother..

[21] Prather A.A., Pressman S.D., Miller G.E., Cohen S. (2021). Temporal Links Between Self-Reported Sleep and Antibody Responses to the Influenza Vaccine. Int. J. Behav. Med..

[22] Benedict C., Brytting M., Markström A., Broman J.E., Schiöth H.B. (2012). Acute sleep deprivation has no lasting effects on the human antibody titer response following a novel influenza A H1N1 virus vaccination. BMC Immunol..

[23] Quach H.Q., Warner N.D., Ovsyannikova I.G., Covassin N., Poland G.A., Somers V.K., Kennedy R.B. (2023). Excessive daytime sleepiness is associated with impaired antibody response to influenza vaccination in older male adults. Front. Cell Infect. Microbiol..

[24] Taylor D.J., Kelly K., Kohut M.L., Song K.S. (2017). Is Insomnia a Risk Factor for Decreased Influenza Vaccine Response?. Behav. Sleep Med..

[25] Wyse C.A., Rudderham L.M., Nordon E.A., Ince L.M., Coogan A.N., Lopez L.M. (2024). Circadian Variation in the Response to Vaccination: A Systematic Review and Evidence Appraisal. J. Biol. Rhythm..

[26] Hazan G., Duek O.A., Alapi H., Mok H., Ganninger A., Ostendorf E., Gierasch C., Chodick G., Greenberg D., Haspel J.A. (2023). Biological rhythms in COVID-19 vaccine effectiveness in an observational cohort study of 1.5 million patients. J. Clin. Investig..

[27] Meyer N., Harvey A.G., Lockley S.W., Dijk D.J. (2022). Circadian rhythms and disorders of the timing of sleep. Lancet.

[28] Han S.H., Lee S.Y., Cho J.W., Kim J.H., Moon H.J., Park H.R., Cho Y.W. (2023). Sleep and Circadian Rhythm in Relation to COVID-19 and COVID-19 Vaccination-National Sleep Survey of South Korea 2022. J. Clin. Med..

[29] Lange T., Dimitrov S., Bollinger T., Diekelmann S., Born J. (2011). Sleep after vaccination boosts immunological memory. J. Immunol..

[30] Lange T., Perras B., Fehm H.L., Born J. (2003). Sleep enhances the human antibody response to hepatitis A vaccination. Psychosom. Med..

[31] Jaiswal S.J., Gadaleta M., Quer G., Radin J.M., Waalen J., Ramos E., Pandit J., Owens R.L. (2024). Objectively measured peri-vaccination sleep does not predict COVID-19 breakthrough infection. Sci. Rep..

[32] Rayatdoost E., Rahmanian M., Sanie M.S., Rahmanian J., Matin S., Kalani N., Kenarkoohi A., Falahi S., Abdoli A. (2022). Sufficient sleep, time of vaccination, and vaccine efficacy: A systematic review of the current evidence and a proposal for COVID-19 vaccination. Yale J. Biol. Med..

[33] Lai F., Li B., Mei J., Zhou Q., Long J., Liang R., Mo R., Peng S., Liu Y., Xiao H. (2023). The impact of vaccination time on the antibody response to an inactivated vaccine against SARS-CoV-2 (IMPROVE-2): A randomized controlled trial. Adv. Biol..

[34] Zhang H., Liu Y., Liu D., Zeng Q., Li L., Zhou Q., Li M., Mei J., Yang N., Mo S. (2021). Time of day influences immune response to an inactivated vaccine against SARS-CoV-2. Cell Res..

[35] Wang W., Balfe P., Eyre D.W., Lumley S.F., O’Donnell D., Warren F., Crook D.W., Jeffery K., Matthews P.C., Klerman E.B. (2022). Time of day of vaccination affects SARS-CoV-2 antibody responses in an observational study of healthcare workers. J. Biol. Rhythm..

[36] Loef B., Dollé M.E.T., Proper K.I., van Baarle D., Initiative L.C.R., van Kerkhof L.W. (2022). Night-shift work is associated with increased susceptibility to SARS-CoV-2 infection. Chronobiol. Int..

[37] Otasowie C.O., Tanner R., Ray D.W., Austyn J.M., Coventry B.J. (2022). Chronovaccination: Harnessing circadian rhythms to optimize immunisation strategies. Front. Immunol..

[38] Schmitz N.C.M., van der Werf Y.D., Lammers-van der Holst H.M. (2022). The Importance of Sleep and Circadian Rhythms for Vaccination Success and Susceptibility to Viral Infections. Clocks Sleep.

[39] Menghini G.M., Thurnheer R., Kahlert C.R., Kohler P., Grässli F., Stocker R., Battegay M., Vuichard-Gysin D. (2024). Impact of shift work and other work-related factors on anti-SARS-CoV-2 spike-protein serum concentrations in healthcare workers after primary mRNA vaccination—A retrospective cohort study. Swiss Med. Wkly..

[40] Brouwers T.M.J., Çobanoğlu Ü.G., Geers D., Rietdijk W.J.R., Gommers L., Bogers S., Lammers G.J., van der Horst G.T.J., Chaves I., GeurtsvanKessel C.H. (2024). The effect of sleep and shift work on the primary immune response to messenger RNA-based COVID-19 vaccination. J. Sleep. Res..

[41] Pigazzani F., Dyar K.A., Morant S.V., Vetter C., Rogers A., Flynn R.W.V., Rorie D.A., Mackenzie I.S., Cappuccio F.P., Manfredini R. (2024). Effect of timed dosing of usual antihypertensives according to patient chronotype on cardiovascular outcomes: The Chronotype sub-study cohort of the Treatment in Morning versus Evening (TIME) study. EClinicalMedicine.

[42] Athanasiou N., Baou K., Papandreou E., Varsou G., Amfilochiou A., Kontou E., Pataka A., Porpodis K., Tsiouprou I., Kaimakamis E. (2023). Association of sleep duration and quality with immunological response after vaccination against severe acute respiratory syndrome coronavirus-2 infection. J. Sleep Res..

[43] Opp M.R. (2023). Sleep: Not getting enough diminishes vaccine responses. Curr. Biol..

[44] Mok H., Ostendorf E., Ganninger A., Adler A.J., Hazan G., Haspel J.A. (2024). Circadian immunity from bench to bedside: A practical guide. J. Clin. Investig..

[45] Ruiz F.S., Rosa D.S., Zimberg I.Z., Dos Santos Quaresma M.V., Nunes J.O., Apostolico J.S., Weckx L.Y., Souza A.R., Narciso F.V., Fernandes-Junior S.A. (2020). Night shift work and immune response to the meningococcal conjugate vaccine in healthy workers: A proof of concept study. Sleep Med..

[46] Ince L.M., Barnoud C., Lutes L.K., Pick R., Wang C., Sinturel F., Chen C.S., de Juan A., Weber J., Holtkamp S.J. (2023). Influence of circadian clocks on adaptive immunity and vaccination responses. Nat. Commun..

[47] Relan P., Motaze N.V., Kothari K., Askie L., Le Polain O., Van Kerkhove M.D., Diaz J., Tirupakuzhi Vijayaraghavan B.K. (2023). Severity and outcomes of Omicron variant of SARS-CoV-2 compared to Delta variant and severity of Omicron sublineages: A systematic review and metanalysis. BMJ Glob. Health.

